# Targeting MCL-1 protein to treat cancer: opportunities and challenges

**DOI:** 10.3389/fonc.2023.1226289

**Published:** 2023-07-31

**Authors:** Shady I. Tantawy, Natalia Timofeeva, Aloke Sarkar, Varsha Gandhi

**Affiliations:** ^1^ Department of Experimental Therapeutics, The University of Texas MD Anderson Cancer Center, Houston, TX, United States; ^2^ Department of Leukemia, The University of Texas MD Anderson Cancer Center, Houston, TX, United States

**Keywords:** MCL-1 protein, BCL-2 family proteins, MCL-1 inhibitors, apoptosis, BCL-2 family, cancer therapy

## Abstract

Evading apoptosis has been linked to tumor development and chemoresistance. One mechanism for this evasion is the overexpression of prosurvival B-cell lymphoma-2 (BCL-2) family proteins, which gives cancer cells a survival advantage. *Mcl-1*, a member of the BCL-2 family, is among the most frequently amplified genes in cancer. Targeting myeloid cell leukemia-1 (MCL-1) protein is a successful strategy to induce apoptosis and overcome tumor resistance to chemotherapy and targeted therapy. Various strategies to inhibit the antiapoptotic activity of MCL-1 protein, including transcription, translation, and the degradation of MCL-1 protein, have been tested. Neutralizing MCL-1’s function by targeting its interactions with other proteins *via* BCL-2 interacting mediator (BIM)_S_2A has been shown to be an equally effective approach. Encouraged by the design of venetoclax and its efficacy in chronic lymphocytic leukemia, scientists have developed other BCL-2 homology (BH3) mimetics—particularly MCL-1 inhibitors (MCL-1i)—that are currently in clinical trials for various cancers. While extensive reviews of MCL-1i are available, critical analyses focusing on the challenges of MCL-1i and their optimization are lacking. In this review, we discuss the current knowledge regarding clinically relevant MCL-1i and focus on predictive biomarkers of response, mechanisms of resistance, major issues associated with use of MCL-1i, and the future use of and maximization of the benefits from these agents.

## Introduction

The process of programmed cell death, apoptosis, is essential for cellular homeostasis, and its dysregulation has been incriminated in tumorigenesis and chemoresistance. Classically, apoptosis is triggered either intrinsically through the mitochondrial pathway or extrinsically through the death ligand-receptor pathway. The former is controlled by the BCL-2 family proteins that include antiapoptotic and proapoptotic molecules (1, 2). Myeloid cell leukemia-1 (MCL-1) is an anti-apoptotic protein that heterodimerizes with the proapoptotic members to prevent apoptotic cell death. MCL-1 is overexpressed in several cancers and mediates resistance to apoptosis triggered by chemotherapy and targeted therapy (3, 4). The development of MCL-1i inhibitors (MCL-1i) has been challenging owing to the shallow BCL-2 homology (BH3) binding groove of the MCL-1 protein (5). Using structure-based drug design, potent MCL-1i with *in vivo* activity have been developed and are now in clinical trials. Efforts are being made to identify biological markers of response or resistance to optimize and maximize the effect of the MCL-1i.

## BCL-2 family proteins regulate apoptosis

Apoptosis is a genetically programmed cell death that is important for development, tissue homeostasis, and immunity. Its dysregulation has been linked to various ailments in which uncontrolled apoptosis can contribute to neurodegenerative diseases, and decreased apoptosis can promote cancer and autoimmune diseases. Apoptosis can be triggered by 2 distinct routes: the mitochondrial and death receptor pathways. Both pathways eventually activate the effector caspases (caspases 3, 7, and 6) to initiate apoptosis (1, 2, 6).

The interactions between different BCL-2 family protein subgroups set the apoptotic threshold for the cells (7, 8). The increase in the BH3-only proapoptotic proteins (initiators) initiates apoptosis by binding to and inactivating the prosurvival proteins (guardians), thereby enabling activation of the proapoptotic effector proteins (BAK, BAX). BAX and BAK homo-oligomerize to disrupt the outer mitochondrial membrane, leading to the release of cytochrome c and second mitochondria-derived activator of caspase (SMAC) from the mitochondria. Cytochrome c activates caspase 9 on the scaffold protein apoptotic protease-activating factor 1 (APAF1), whereas SMAC blocks the caspase inhibitor X-linked inhibitor of apoptosis protein (XIAP) (9, 10). This eventually leads to caspase-mediated cleavage of cellular proteins and the initiation of cell death.

The BCL-2 family proteins share 1 or more of the 4 characteristic BH domains (designated as BH1, BH2, BH3, and BH4) (11). A common general structure in the BCL-2 family proteins is a central hydrophobic α-helix surrounded by amphipathic α-helices (12). These α-helices form a tertiary structure with a hydrophobic BH3 domain–binding groove that can bind to the BH3 domains of other family members. C- terminus transmembrane domains help some members to localize to the mitochondria. BCL-2 proteins can be classified into antiapoptotic proteins (BCL-2, MCL-1, BCL-extra-large [BCL-xL], BFL-1/BCL-2–related protein A1 [BCL-2A1], BCL-W, BCL-B), multidomain proapoptotic executioner proteins (BAX, BAK, and BOK) and BH3-only proapoptotic proteins (BIM, BAD, PUMA, Noxa, HRK, and BMF) (13). BH3-only proteins show specificity towards targeting their prosurvival partners. While BIM, PUMA, and BID act as nonselective general binders to prosurvival proteins, BAD specifically binds BCL-2, BCL-xL, and BCL-W. On the other hand, Noxa selectively binds to MCL-1 and BCL-2A1 (14–16).

## The *Mcl-1* gene and its expression

In 1993, Kozopas et al. (17) identified *Mcl-1* while exploring the genes that are induced by phorbol 12-myristate 13-acetate. The *Mcl-1* gene is homologous to the BCL-2 gene. It is located at 1q21.2 with a promotor region that has several transcription factors binding sites; these include, but are not limited to, STAT, cAMP response elements, and nuclear factor kappa B binding sites. *Mcl-1* is among the most amplified genes in cancer. A subset of multiple myeloma patients (40%) had amplification or gain of *Mcl-1* gene (1q21) (18), with significantly shorter progression-free survival and lower overall survival (19). When 3000 samples from 26 different cancers were profiled, the *Mcl-1* and *BCL-2A1* genes were among the most amplified genes (3). Amplification was relatively higher in breast and non-small cell lung cancer (NSCLC) than in the other types of cancer and was correlated with higher amounts of *Mcl-1* messenger RNA (mRNA) and unfavorable overall survival in patients (20).

The *Mcl-1* gene encodes the full-length MCL-1 protein (MCL-1L, isoform 1) with antiapoptotic function. The alternatively spliced, shorter gene products (MCL-1S, isoform 2 and isoform 3) have a proapoptotic function, as they bind and inactivate MCL-1L (21–23). Switching from antiapoptotic to proapoptotic MCL-1 protein by controlling MCL-1 splicing appears to be a promising strategy in cancer therapy (24). Accordingly, splicing factor 3B subunit 1 (SF3B1) inhibitors have been recently explored as sensitizers that can switch on alternative splicing to generate proapoptotic MCL-1S isoforms (25). The MCL-1 transcript has adenylate-uridylate–rich elements that control the rapid turnover and short half-life of *Mcl-1* mRNA (26).

## MCL-1 protein: structure; regulation; and function

**Figure 1:**
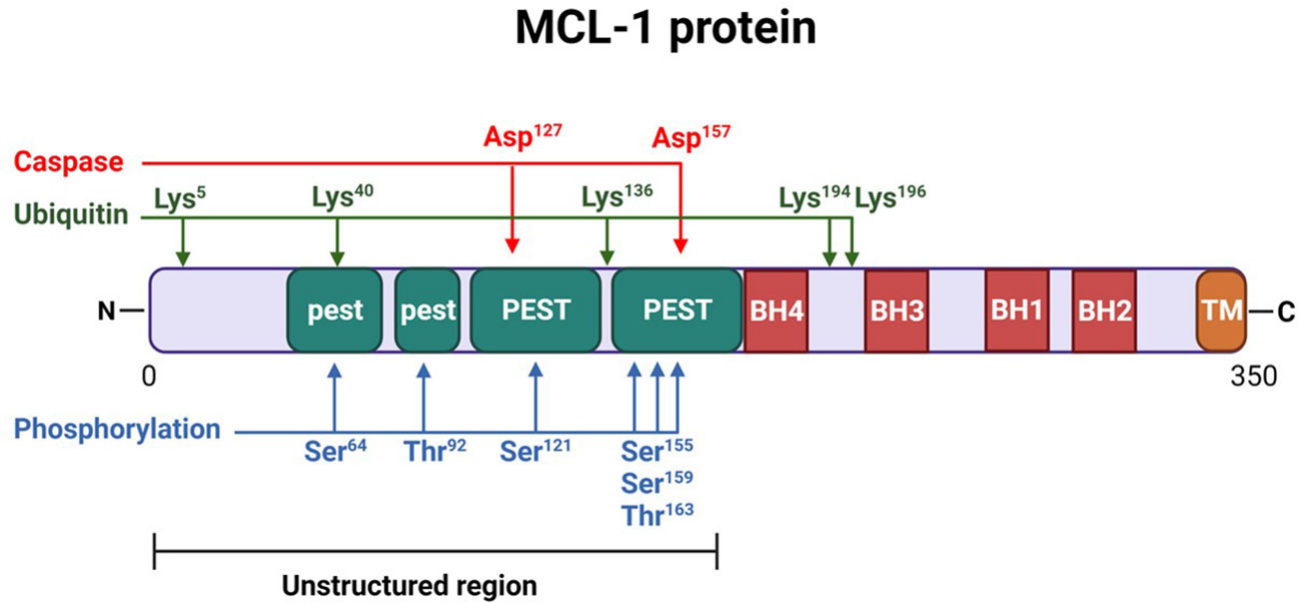
Structure of myeloid cell leukemia-1 (MCL-1) protein. MCL-1 protein consists of 350 amino acids, highlighting several important post-translational modification sites. Two caspase cleavage sites (aspartic acid [Asp] 127 and 157), 5 lysine [Lys] residues (5, 40, 136, 194, and 196) for ubiquitination, and 6 phosphorylation sites (serine [Ser] 64, threonine [Thr] 92, Ser 121, 155, and 159, and Thr 163) are indicated. The N-terminus of the protein is largely unstructured and includes 4 proline, glutamate, serine, and threonine (PEST) regions (2 major and 2 minor, labeled as “PEST” and “pest,” respectively). Four BCL-2 homology (BH) domains (BH1-BH4) are located near the C-terminus, which also contains the transmembrane (TM) domain necessary for mitochondrial localization.

MCL-1 protein is a unique member of the BCL-2 family of proteins. It is larger than its other prosurvival relatives (350 amino acid residues [37 kd] versus 239 residues for BCL-2 and 233 residues for BCL-xL). The C-terminus of MCL-1 protein (170-350 aa) has a transmembrane domain that is important for the mitochondrial localization of MCL-1 protein (27) (**Figure 1**). It also harbors 4 BH domains that regulate MCL-1’s interaction with other proteins (28). The BH3 domain of MCL-1 has a binding groove that interacts with other BH3-domain proteins (Noxa, BIM, BAK). This binding groove has four pockets (P1-P4) that binds to hydrophobic side chains (H1-H4) of the proapoptotic proteins (5, 29). In addition, the BH3 domain harbors the QRN motif that controls MCL-1 ubiquitination and degradation. The BH1 and BH3 domains also interact with KU70/KU80 dimers to repair DNA. The N-terminus of MCL-1 (1-170 residues) is long and unstructured, and it is absent in other BCL-2–related survival proteins. The N-terminus is rich in multiple proline (P), glutamate (E), serine (S), and threonine (T) residues (PEST regions) that regulate MCL-1’s stability and function (17) and result in rapid turnover and are characteristic of short-half-life proteins like MCL-1. These sequences also contain mitochondrial targeting signals.

The regulation of MCL-1 has been studied extensively (30–33), and its expression is controlled at the transcriptional, post-transcriptional, translational, and post-translational levels. Several modulators regulate MCL-1 protein levels in response to both internal and external stimuli such as cytokines and growth factors; these modulators induce signal transduction and the activation of transcription factors (34), endoplasmic reticulum stress (35), hypoxia (36), and microRNAs (37). Several post-translational modifications—including phosphorylation mediated by c-Jun N-terminal kinase, glycogen synthase kinase 3β (GSK-3β), and extracellular signal-regulated kinase (ERK)-1; ubiquitination (Mule, SCFβ-TrCP, SCFFbw7, APC/CCdc20, and Trim17 E3 ubiquitin ligases) and deubiquitination (USP9x); and caspase-mediated cleavage (38)—regulate the stability and functional activity of MCL-1. In addition, the interaction of MCL-1 protein with other BH3-only proteins can affect the stability and proteasomal degradation of MCL-1 protein. While BIM and PUMA binding leads to the stabilization of MCL-1 protein, Noxa binding enhances the degradation of MCL-1 protein (39, 40). MCL-1 is widely expressed in human tissue, including the bone marrow, lymph nodes, spleen, gall bladder, appendix, and heart (21, 22, 41, 42). In summary, MCL-1 is an early-response gene, and the presence of adenylate-uridylate–rich elements in transcripts and PEST domains in proteins result in fast transcript and protein turnover.

MCL-1 protein has antiapoptotic function that is important for cell viability. It inhibits apoptosis by binding and sequestering multidomain BH effector proteins (e.g., BAK, BAX), thus preventing their activation and mitochondrial outer membrane permeabilization. As mentioned before, MCL-1 protein binds preferentially to BAK and Noxa, while BIM and PUMA bind to all prosurvival proteins (43). MCL-1 is located in the nucleus, where it controls cell cycle progression and helps with DNA repair, and in the mitochondria, where it regulates other functions. Besides sequestering BAK, MCL-1 helps mitochondrial fragmentation by recruiting Drp1 and mitochondrial fusion *via* the stabilization of OPA1 in the inner mitochondrial membrane (44). MCL-1 also supports mitochondrial metabolic activities, it directly engages very-long-chain acyl-CoA dehydrogenase, preventing its stress induced excessive activity (45). It also promotes oxidative phosphorylation through uncharacterized mechanisms (46). MCL-1 inhibits beclin-1, and thus it can regulate autophagy. Its effect on autophagy depends on the cellular context. It also inhibits mitophagy through parkin/pink1, independently of beclin-1 (47). The role of MCL-1 in mitophagy and mitochondrial function may allow MCL-1 to be used therapeutically to target neurodegenerative diseases, including Alzheimer’s disease (48, 49). MCL-1 also has a role in cell senescence. In 2015, Demelash et al. (50) identified a loop domain responsible for inhibiting cellular senescence induced by chemotherapy, thus providing resistance to cancer therapy. MCL-1 also has an established role in the repair of DNA double-strand breaks (51). Collectively, these findings suggest a multidimensional role for MCL-1 protein.

## MCL-1 as a target for cancer therapy

As mentioned earlier, *Mcl-1* gene amplification was highest among many tumors. Similarly, MCL-1 protein has been implicated in both tumorigenesis and chemotherapeutic resistance (4, 52). Consistent with this statement, it has been shown that, in xenograft models, knockdown of MCL-1 decreased the proliferation rate of cancer cells to a greater degree than that seen in controls (3). Conversely, increased incidence of B-cell lymphoma was noticed in transgenic mice overexpressing MCL-1 (53).

MCL-1 overexpression has been observed in several hematological and solid tumors (54–59). In clinical studies, multiple myeloma (MM) patients with high MCL-1 level had shorter event free survival (60). Similarly, MM patients overexpressing USP9x, which leads to increased MCL-1 stability, were shown to have a poor prognosis and can promote tumor survival (61). It has been shown that high levels of MCL-1 expression are required for B-lymphoma cell survival (62) and correlate with high-grade lymphoma (63), suggesting an association between high levels of MCL-1 expression and progressive disease (64). Interestingly, in glioblastoma, somatic mutations in the PEST region of MCL-1 (D155G, D155H, and L174S) were shown to stabilize MCL-1; these mutations may participate in gliomagenesis. Indeed, overexpressing these mutant plasmids in glioma cells accelerated the growth of glioma cells compared to wild type (WT) MCL-1 cells (65).

MCL-1 overexpression has also been implicated in resistance to both targeted therapy (such as venetoclax and navitoclax) (66–68) and conventional chemotherapy, including taxol, cisplatin, erlotinib, and cytarabine (69–71). In 2022, Zhang et al. (72) showed that there was selection for RAS-mutant clones in patients with acute myelogenous leukemia (AML) treated with venetoclax. These clones mediated resistance to venetoclax through MCL-1 upregulation, and cells were resensitized to the drug through the inhibition of MCL-1. Besides, concomitant use of MCL-1 inhibitors and venetoclax was better than other combination regimens in venetoclax resistant models (73). The overexpression of MCL-1 is related to cisplatin resistance (74). Depleting MCL-1 has reversed resistance to cisplatin and doxorubicin in osteosarcoma cell lines *in vitro* and xenograft tumors *in vivo* (75). MCL-1 amplification has been found in subsets of wild-type fibroblast growth factor receptor urothelial cancer, and its degradation by erdafitinib synergized BCL-xL/BCL-2 inhibitors (76).

Also, it has been shown that the knockdown of USP9X and expression of FBW7 in FBW7-deficient cells leads to increased MCL-1 protein turnover and sensitizes cells to ABT-737, suggesting the role of MCL-1 in the resistance to BCL-2/BCL-xl inhibitors (61, 77). MCL-1 inhibits chemotherapy induced senescence, thus mediating resistance to cancer therapy (50). MCL-1 is upregulated in senescent tumor cells and in cells that express low levels of BCL-2. While the BCL-2 inhibitor navitoclax successfully reduced metastases in mice with tumors, the MCL-1 inhibitor S63845 completely eradicated both senescent tumor cells and metastases (78). The direct inhibition of MCL-1 protein by AZD5991 or indirectly by CDK9 inhibitor (AZD4573), can overcome venetoclax resistance in AML cell lines and PDXs models (79). Besides, downregulating MCL-1 using PIK-75, a dual PI3K and CDK9 inhibitor, overcome venetoclax resistance in MCL cell lines (80).

## Targeting MCL-1

**Figure 2:**
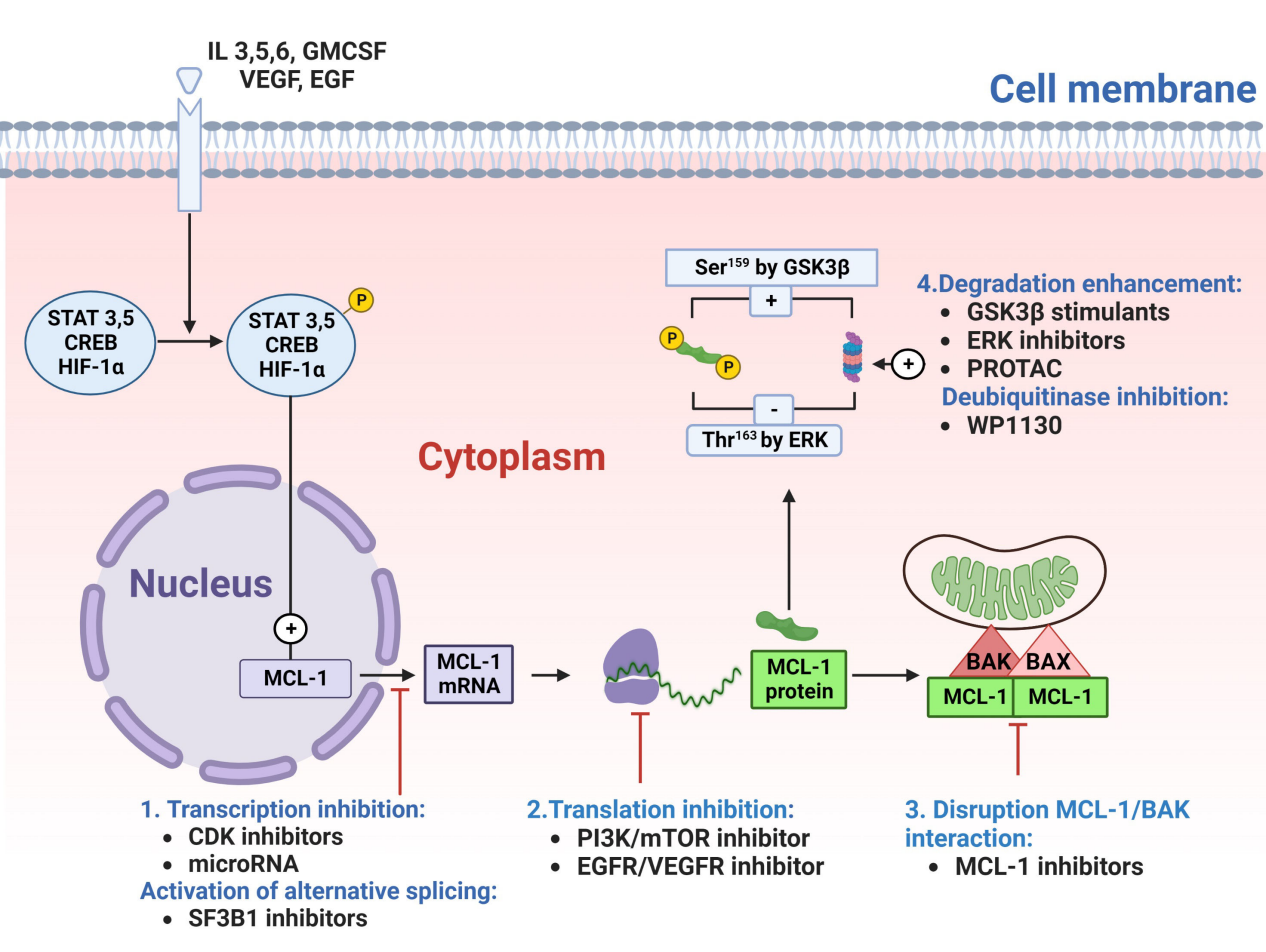
Targeting myeloid cell leukemia-1 (MCL-1) protein. MCL-1 protein can be targeted either directly, by disrupting MCL-1 interaction with proapoptotic proteins (#3), or indirectly by targeting MCL-1 protein at stages of transcription(#1), translation(#2) or degradation(#4). Cyclin-dependent kinase (CDK)7/9 inhibitors or microRNA can block the transcription of MCL-1 messenger RNA (mRNA). SF3B1 inhibitors can generate a proapoptotic MCL-1S isoform by switching on alternative splicing. Once transcribed, MCL-1 mRNA moves to the cytoplasm for translation into MCL-1 protein (depicted in green). This translation process can be prevented with phosphatidylinositol-3 kinase (PI3K)/mammalian target of rapamycin (mTOR) inhibitors or epidermal growth factor receptor (EGFR)/vascular endothelial growth factor receptor (VEGFR) inhibitors. After translation, MCL-1 protein binds and deactivates the BAK/BAX complex (depicted in red/pink) in the outer mitochondrial membrane. This binding can be disrupted with direct MCL-1 inhibitors), leading to BAX/BAK dissociation and apoptosis. Additionally, the proteasomal degradation of MCL-1 can be enhanced using glycogen synthase kinase 3β (GSK3β) stimulants, extracellular signal-regulated kinase (ERK) inhibitors, the proteolysis-targeting chimera (PROTAC), or deubiquitinating enzymes (DUB) inhibitors (e.g., WP1130), thereby increasing MCL-1 polyubiquitination and subsequent degradation. CREB, cAMP response element-binding protein; GMCSF, Granulocyte-macrophage colony-stimulating factor; HIF-1α, Hypoxia-inducible factor 1-alpha; IL, interleukin; Ser, serine; STAT, signal transducer and activator of transcription; Thr, threonine.

MCL-1 protein can be targeted indirectly through modulating *Mcl-1* gene transcription and translation or directly through inhibiting the functional interaction of MCL-1 with BAK (**Figure 2**). The cyclin-dependent kinases (CDKs) 7 and 9 play pivotal roles in the transcription of genes. MCL-1 protein expression has been modulated by inhibiting transcription using CDK 7/9 inhibitors such as flavopiridol, roscovitine, dinaciclib, and SNS-032 (81, 82).

Similar to MCL-1 transcription inhibition, MCL-1 protein synthesis is blocked by inhibitors of protein translation such as omacetaxine. Omacetaxine binds to A-site cleft of ribosomes and is currently approved for the treatment of refractory chronic myelogenous leukemia. Its clinical success in producing cytogenetic and hematologic responses is based on its ability to decrease the oncoproteins Bcr-Abl, MCL-1, and c-Myc (83).

Other inhibitors of protein translation include the PI3K/mTOR inhibitors (BEZ235 and AZD8055), EGFR/VEGFR inhibitors (BAY43-9006), elF4F inhibitors (Silvestrol) or dephosphorylation of eukaryotic initiation factor 4G (benzyl isothiocyanate) (84–87). Also, MCL-1 protein can be targeted for proteasomal degradation by stimulating the GSK3β enzyme (with arsenic trioxide or bufalin) (88, 89), inhibiting the MEK/ERK pathway (with trametinib) (90), treating with deubiquitinase inhibitor WP1130 (91), or inducing selective intracellular proteolysis of MCL-1 protein *via* the proteolysis-targeting chimera (PROTAC) strategy (92). MCL-1 can also be targeted for degradation by upregulating Noxa. Alternatively, MCL-1 interaction can be targeted by the allosteric inhibition of MCL-1 protein (93) and direct inhibition of the MCL-1 protein-BH3 domain interaction (**Figure 2**).

## Optimizing the design of MCL-1 inhibitors

Lee et al. (94) were the first researchers to target MCL-1 protein-protein interaction and to show that MCL-1 neutralization is an effective approach to MCL-1 targeting. This was an important observation for the development of small-molecule drugs directly targeting MCL-1.

Since then, researchers have focused on developing specific and potent MCL-1i. This work required overcoming 3 major challenges, that delayed the development of direct inhibitors of MCL-1. First, MCL-1’s shallow and relatively inflexible binding groove has delayed the development of high affinity MCL-1i. Besides, the structure of the BH3-binding grooves is without topological features and is very similar to various proteins of the prosurvival BCL-2 family, making it challenging to achieve selectivity within the family (95–97). Second, there is a lack of specificity to MCL-1 binding; because of this, early MCL-1i (gossypol (98), apogossypolone (99), antimycin A (100), obatoclax (101), and TW-37 (102)) were neither selective nor potent (103). Third, poor pharmacokinetic profiles and limited cell membrane permeability (104) were primary factors excluding MCL-1i from clinical use.

To overcome these challenges, researchers have tried to identify hot spots in MCL-1 protein that are critical for the stable binding of BH3 mimetics to the MCL-1 binding groove. Using nuclear magnetic resonance, X-ray crystallography, and alanine mutagenesis studies, researchers identified 4 hydrophobic pockets (P1-P4) and arginine 263 (Arg263) residue in the MCL-1 BH3 groove as hot spots required for peptide binding (105). Further studies revealed that, compared to BCL-2 and BCL-xL, the P2 and P3 pockets of MCL-1 have the most potential to bind MCL-1–binding peptides (106, 107). The P2 hydrophobic groove is relatively larger than the P3 hydrophobic groove and thus can accommodate ligands with larger structure moiety. Occupying the P3 and P4 pockets could further improve the binding affinity to MCL-1 (108). Additionally, the Arg263 residue was found to be an important hot spot that forms a hydrogen bond (salt bridge) with MCL-1i. Cocrystal structure analysis proved that this salt bridge formation was essential for MCL-1i’s efficacy; thus indole moiety may provide structural privilege for MCL-1i (109–111).

The direct, specific inhibitors of MCL-1’s protein-protein interactions have a similar structure to that of the BH3-only protein motifs and bind to MCL-1’s BH3 hydrophobic groove (112). Based on their structures, these inhibitors can be categorized into different types, including peptide inhibitors, marinopyrrole derivatives, gossypol derivatives, quinoline derivatives, S1 derivatives, and indole derivatives (113). The use of these inhibitors in MCL-1–dependent cell lines disrupted MCL-1:BAK interaction, thus paving the way for proapoptotic BH3 proteins (i.e. BIM) to bind BAK and initiate the intrinsic cell death pathway (5, 114, 115).

Since 2016, as many as 6 MCL-1i, including AMG-176 (114), AMG-397 (116), S64315 (5), AZD5991 (115), ABBV-467, and PRT1419, have been tested in the clinic. S63845 was the first MCL-1i reported to have potent *in vivo* activity and to enter clinical trials (NCT02992483) (5). Several clinical trials were initiated with AZD5991, AMG-176, AMG-397, and S64315 (**Table 1**), mostly in hematological malignancies owing to their preferential dependence on MCL-1 for growth. MCL-1i were tested in phase 1 trials as single agents or in combination with venetoclax or carfilzomib.

**Table 1:** MCL-1 inhibitors in clinical trials.

MCL-1i	Study	Status	Conditions studied	Interventions	Clinical trial
AZD5991	Phase 1/2 study of AZD5991 in R/R hematologic malignancies	Terminated	R/R hematologic malignancies (NHL, CLL, Richter transformation, TCL, MM,AML, MDS)	AZD5991 (phase 1): Monotherapy with multiple dose levels given *via* IV for 9 cycles (21 days each) or until patients show response or progress. AZD5991 + venetoclax (phase 2): Ascending oral doses of venetoclax until no longer tolerated or disease progresses.	NCT03218683
AMG-176	Phase 1 study of venetoclax and AMG-176 in patients with R/R hematologic malignancies	Terminated	AML/NHL/DLBCL	Oral venetoclax and IV AMG-176 will be administered in different combinations of dose levels.	NCT03797261
	Phase 1 study of AMG-176 alone/in combination with azacytidine in MDS and CMML	Recruiting	MDS/CMML	Dose exploration and dose expansion studies in which AMG-176 will be given *via* IV and azacytidine will be given *via* IV or SC injection.	NCT05209152
	Phase 1 study of AMG-176 in R/R MM and AML	Recruiting	R/R MM, R/R AML	AMG-176 monotherapy in R/R MM or in combination with azacytidine/itraconazole in R/R AML.	NCT02675452
AMG-397	Phase 1 study of the safety, tolerability, pharmacokinetics, and efficacy of AMG 397 in MM, NHL, and AML	Terminated	MM/NHL/AML/DLBCL	AMG-397 administered orally once daily for 2 consecutive days followed by a 5-day break at a weekly interval (part of a 28-day treatment cycle in adult subjects).	NCT03465540
S64315/ MIK665	Phase 1 of study of IV S64315 in combination with oral venetoclax in patients with AML	Active, not recruiting	AML	21-day cycle with weekly IV S64315 (50 mg to 100 mg once a week) and daily oral venetoclax (start at 100 mg and give up to 600 mg daily). S64315 should be administered 2 to 4 hours before venetoclax.	NCT03672695
	Phase 1 study of IV S64315 in patients with AML or MDS	Active, not recruiting	AML/MDS	S64315 will be administered either once weekly (21-day cycle) or twice weekly (28-day cycle) *via* IV infusion over 30 min to 3 hr. The starting dose is 50 mg.	NCT02979366
	Phase 1 study of MIK665 in patients with R/R lymphoma or MM	Recruiting	MM/lymphoma/DLBCL	This study will utilize a Bayesian hierarchical model to guide dose escalation and estimate the MTD(s) based on the dose-DLT relationship(s) for MIK665.	NCT02992483

AML, acute myeloid leukemia; CMML, chronic myelomonocytic leukemia; CLL, chronic lymphocytic leukemia; DLBCL, diffuse large B-cell lymphoma; DLT, dose-limiting toxicity; IV, intravenous; MDS, myelodysplastic syndromes; MM, multiple myeloma; MTD, maximum tolerated dose; NHL, non-Hodgkin lymphoma; R/R, refractory or relapsing; SC, subcutaneous; TCL, T-cell lymphoma.

### A-1210477

A-1210477 (in the indole-2-carboxylic acid group), developed by AbbVie, is the first selective high affinity inhibitor of MCL-1. It binds to MCL-1 with a *K*_i_ value of 0.454 nM, and has been shown to disrupt the MCL-1/BIM interactions that induce apoptosis only in cell lines that show dependency on MCL-1 protein (H929, H2110, and H23) (117). Moreover, it synergized with navitoclax in various cancer cell lines (118) and overcame resistance to navitoclax (119). Also, A-1210477 has been shown to be synergistic when combined with an inhibitor of Hedgehog signaling (120) (i.e., bromodomain extra-terminal protein inhibitor [ABBV-075] in CD34+ AML cells (121) or ABT-263 [navitoclax]) (122). MCL-1 has been shown to bind to and inhibit the transcriptional function of the tumor suppressor p73 through its BH3 domain. Consequently, A-1210477 has been used to induce p73 and thereby activate DNA double-strand break repair target gene expression, promoting cell cycle arrest and apoptosis (123). Because of its low potency, A1210477 remains a tool compound.

### VU661013

VU661013 is a potent MCL-1i that binds to MCL-1 with high affinity and was developed using a fragment-based screening and structure-based design to optimize a previously reported MCL-1i. VU661013 inhibited MCL-1 (*K_i_* 97 ± 30 pmol/L) without significant inhibition of BCL-xL or BCL-2. Accordingly, VU661013 inhibited growth in AML cell lines, except those dependent on BCL-2 (124).

Venetoclax enhanced the cellular cytotoxicity of VU661013, even in AML cell lines and AML blasts with innate and/or acquired resistance to VU661013, when the cell lines or blasts were treated with venetoclax before or venetoclax and low dose cytarabine after VU661013 treatment. Further, an MV-4-11 cell line–based xenograft mouse model for AML showed a dose-dependent decrease in CD45+ MV-4-11 cells in the blood, bone marrow, and spleen; no evidence of toxicity in non-target tissues, and increased survival after VU661013 treatment (66). In addition to being effective in liquid tumors, the drug was effective in estrogen receptor–positive breast cancer cells *in vitro* and in a MCF7 xenograft model (125).

### AZD5991

AZD5991 is a macrocyclic high affinity MCL-1i (K_i_ = 0.2 nM) developed by AstraZeneca from indole-2-carboxylic acids (115). It disrupts BAK/MCL-1 interaction to induce apoptosis, with preferential activities in hematological cancer cell lines and some NSCLC and breast cancer cell lines as well as primary myeloma cells. Besides, AZD5991 showed potent *in vitro* activity against primary leukemia cells and *in vivo* antitumor activity in various AML, non-Hodgkin lymphoma, and MM mouse xenograft models. The activity of AZD5991 against MM and AML in murine models was substantial and was further enhanced with bortezomib or venetoclax, respectively (115).

AZD5991 was also effective in solid tumor cells and demonstrated promise in colorectal carcinoma cell lines when combined with the multi kinase inhibitor, regorafenib (126). In 2019, Koch et al. (127) reported AZD5991’s activity in T-cell lymphoma cell lines and patient-derived xenograft models *in vivo*. In the models dependent on MCL-1, AZD5991 improved survival alone and in combination with cyclophosphamide, doxorubicin hydrochloride, vincristine sulfate, and prednisone (CHOP) chemotherapy. When used in acquired BRAFi + MEKi resistance model, AZD5991 enhanced the efficacy of ERK1/2 inhibitor (128). Interestingly, AZD5991 synergized with CB-839, a glutaminase inhibitor, to induce apoptosis and inhibit proliferation of CLL cell lines (129). The safety and clinical activities of AZD5991 are being investigated in phase 1 clinical trial (NCT03218683).

### AMG-176 and AMG-397

Caenpeel et al. (114) reported the discovery of AMG-176, a nonindole acid MCL-1i, which was identified using a “structure-based design and conformational restriction”. AMG-176 has a high affinity for MCL-1 protein (K_i_ = 0.06 nM) and a minimal binding affinity to BCL-2 and BCL-xL. *In vitro* studies with AM-8621 (a structural analogue of AMG-176) showed that it disrupted MCL-1/BAK interactions, induced BAX/BAK dependent apoptosis, and increased MCL-1 stability in various hematological malignancy cell lines. *In vivo* sensitivity was also observed in a subcutaneous xenograft model of MM and an orthotopic model of AML (114). AMG-176 treatment decreased peripheral blood and bone marrow cells including B cells, monocytes, neutrophils, eosinophils, basophils, and reticulocytes, which can be used as a pharmacodynamic endpoints to assess treatments (114).

In various models, AMG-176 has shown synergism in combination with different drugs (114). Similar to AZD5991, AMG-176 was also synergistic with carfilzomib (115). Moreover, the combination of venetoclax and AMG-176 was synergistic in AML orthotopic model and in *ex vivo* primary AML patient samples (114) and CLL primary lymphocytes (130). While AMG-176 showed robust antitumor activity in liquid neoplasms, it only showed modest antitumor activity in a few solid tumor cell lines (114).

The safety and clinical activity of AMG-176 is being evaluated in patients with relapsed or refractory MM or AML (NCT02675452). In addition, intervention studies on AMG-176, azacytidine, and itraconazole (NCT02675452) in patients with relapsed/refractory myeloma or AML are ongoing (131). A clinical trial is also evaluating AMG-397 (NCT03465540), the first MCL-1i given orally in the clinic. The use of AMG-397 for the treatment of various blood malignancies has been placed on clinical hold recently following incidences of cardiac toxicity (132).

### S63845 and S64315/MIK665

S63845 is the first selective MCL-1i reported to have *in vivo* activity (5); it induced apoptosis in various hematological cell lines (MM, lymphoma, chronic myelogenous leukemia, AML, and T-cell acute lymphoblastic leukemia [T-ALL]) with half-maximal inhibitory concentration (IC_50_) values less than 100 nM (5, 133, 134). S63845 also showed potent antitumor activity in MM, lymphoma, and AML *in vivo* models (5). In CLL patient samples, S64315 induced apoptosis in a dose-dependent manner (135).

Venetoclax enhanced the S63845-induced apoptosis in AML in *in vivo* and *in vitro* models and in primary AML patient samples. This effect was even better than that for the combination of S63845 and standard-of-care AML chemotherapy, such as decitabine, idarubicin, and cytarabine (136). Similar findings were seen in T-ALL cell lines and in a zebrafish model of T-ALL (133). On the other hand, most solid tumors were resistant to S63845; only a few NSCLC, breast cancer, and melanoma cell lines had a modest response to the drug (5). In triple negative breast cancer, S63845 increased antitumor activity when combined with either chemotherapy or human epidermal growth factor receptor 2 (HER2)-targeted therapies (lapatinib, trastuzumab) (137).

S63845 had limited activity when tested in mice in initial studies. This was attributed to the lower affinity of S63845 to mouse MCL-1 (5). However, in human MCL-1 knock-in models, S63845 was tolerable and effective (138). S63845 was not selected for clinical evaluation, but second-generation S64315 (MIK665) was selected for clinical use in various hematological malignancies (NCT02979366 and NCT02992483) (**Table 1**).

### PRT1419 and ABBV-467

PRT1419 is a new MCL-1i developed by Prelude Therapeutics. When given as a monotherapy in preclinical mouse models of MM, AML, and diffuse large B-cell lymphoma (DLBCL), and when given in combination with venetoclax in an AML model, it caused significant tumor regression (139). This agent is currently in clinical trials for patients with relapsed/refractory (R/R) hematologic malignancies, including lymphoid and myeloid disorders (NCT04543305), or with advanced solid tumors (NCT04837677), including melanoma, sarcoma, breast cancer, and lung cancer. It is also being tested in combination with either azacytidine or venetoclax in R/R myeloid and B-cell malignancies (NCT05107856). Additionally, AbbVie developed ABBV-467, which has been tested as monotherapy for MM patients (NCT04178902) in a phase 1 clinical trial. However, the trial has been terminated.

## MCL-1 inhibitors and upregulation of MCL-1 protein

Interestingly, the MCL-1i upregulate MCL-1 protein in cell lines and in primary patient samples. This finding has not been seen with other BH3 mimetics that target BCL-2 or BCL-xL. Fluorescence resonance energy transfer studies have confirmed that this upregulation correlates with engagement of the MCL-1i into the MCL-1 protein. Thus, MCL-1 upregulation can be considered as a biomarker for MCL-1i target engagement (115).

**Figure 3:**
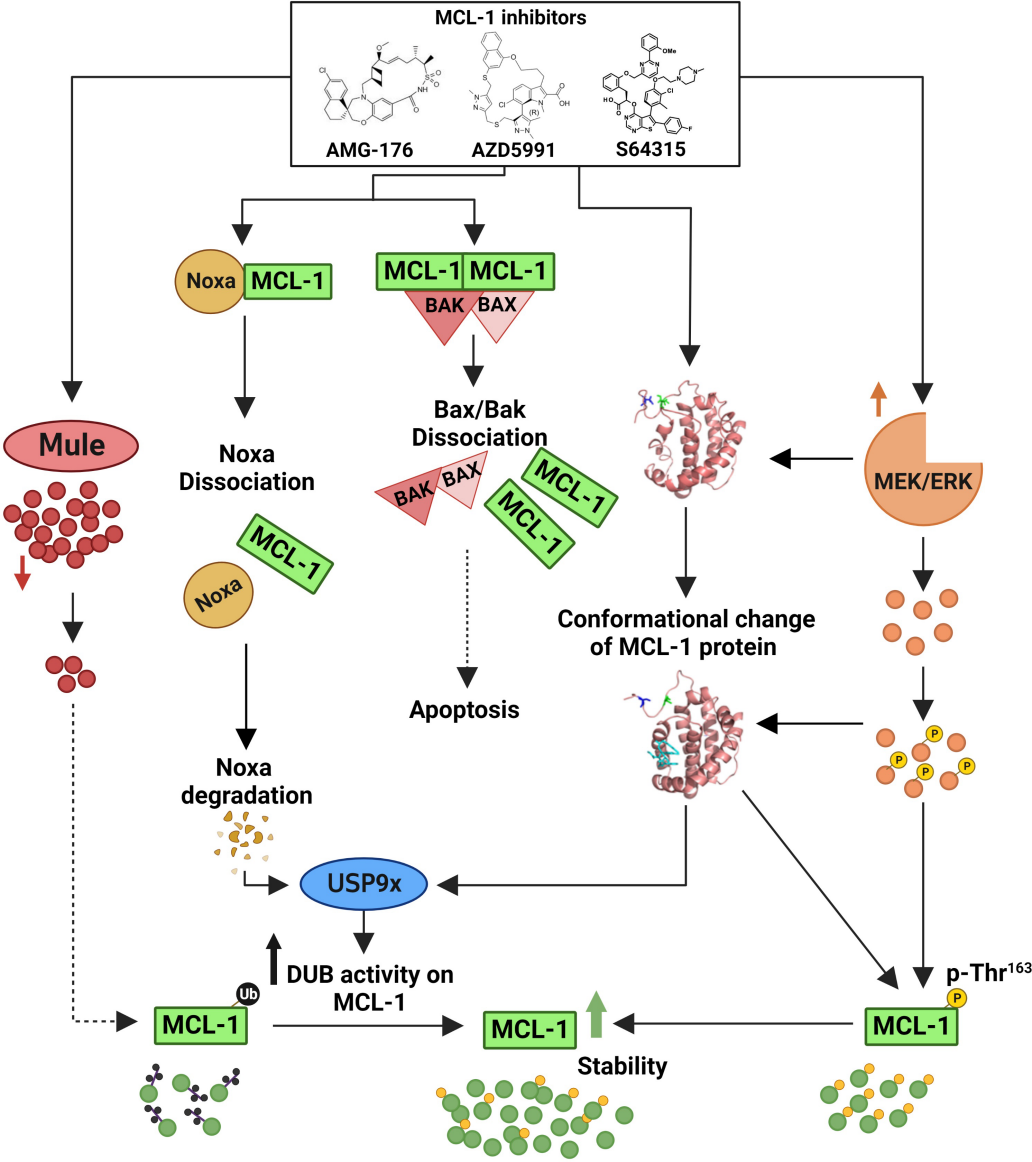
Mechanisms of myeloid cell leukemia-1 (MCL-1) protein upregulation by MCL-1 inhibitors. MCL-1i bind to the BCL-2 homology 3 (BH3) domain of MCL-1 and directly induce a conformation change/a state of MCL-1 that favors deubiquitination by deubiquitinating enzymes (DUBs [e.g., USP9x] rather than ubiquitination. This deubiquitination is further enhanced by Noxa disruption and rapid degradation, leading to enhanced DUBs activity on the MCL-1 protein. Additionally, the binding of MCL-1i transiently decreases the expression of the E3 ligase Mule and increases MEK/ERK-mediated threonine (Thr)163 phosphorylation of MCL-1, thus further contributing to the observed protein stability. Despite this upregulation of MCL-1 protein, MCL-1i disrupted the MCL-1–BAK/BAX interaction to induce apoptosis. The exact mechanism for MCL-1 upregulation was not studied using S63415, however, it is predicted to be the same owing to similar structure and function. Figure is adapted from Tantawy, et al, Clinical Cancer Research).

This upregulation was related to the increased stability of MCL-1 rather than to increased transcription. Recently, Tantawy et al. (140) showed that the MCL-1i-induced stability of MCL-1 protein is mainly due to defective ubiquitination of MCL-1 (**Figure 3**). They showed that MCL-1i directly induced a state of MCL-1 that does not favor ubiquitination; rather, it favors deubiquitination by USP9x. The researchers also observed downregulation of Noxa and a transient decrease in the E3 ligase Mule following treatment with MCL-1i. The use of the deubiquitinase inhibitor WP1130 completely abrogated MCL-1 induction, reaffirming a critical role for deubiquitinases in stabilizing MCL-1 in response to MCL-1i (140). Despite the disruption of the BH3 proapoptotic members (e.g., BAK and Noxa) following MCL-1i treatment, the BH3 E3 ligase Mule was not disrupted. This finding was also seen with S64315 (141), AMG-176, and AZD5991 (140). Thus, MCL-1i can also be considered as anti-Noxa. Despite this upregulation of MCL-1, it did not confer any resistance to the MCL-1i. Besides decreased ubiquitination of MCL-1, another post-translation modification was noticed; MCL-1i induced ERK-mediated phosphorylation of Thr163 MCL-1 (140, 142). The effect of this upregulation and post-translation modification on the non-antiapoptotic function of MCL-1 needs to be elucidated.

## Similarities and differences between MCL-1 inhibitors

Most of the small molecule inhibitors of MCL-1 were discovered using structure-based design followed by fragment-based screening (143). This long and challenging pathway of optimizing MCL-1i has led S63845, AMG-176 and AZD5991 to be clinical candidates. Unlike AZD5991 and S63845, the AMG-176 compound lacks the salt bridge formation with MCL-1 Arg263 residue, and the chlorine atom in AMG-176 is buried in the hydrophobic pocket and does not interact with Ala227 (144). Understanding this difference is critical in the development of MCL-1i, as a recent study in CLL revealed that AZD5991 was more potent than AMG-176 in inducing apoptosis in primary CLL patient samples and the Mino cell line (140). Also, despite MCL-1 stabilization in cell lines following treatment with MCL-1i, cell-free *in vitro* ubiquitination studies revealed that, unlike AZD5991, AMG-176 induced *in vitro* ubiquitination of MCL-1 protein even in the absence of Mule (140). This observation raised 2 important questions. First, can the difference between AMG-176 and AZD5991 in binding MCL-1 protein explain their differences in potency in CLL, given that the salt bridge formation with Arg263 has been shown to be critical for the efficacy for some MCL-1i? Second, can the behavior of MCL-1i in cell-free *in vitro* ubiquitination assays (whether favoring or not favoring MCL-1 ubiquitination) predict the MCL-1i’s efficacy or potency, assuming that potent MCL-1i will behave similarly to AZD5991 in not favoring *in vitro* ubiquitination?

## Maximizing the impact of MCL-1 inhibitors

New approaches to MCL-1 inhibition-based therapy have focused on enhancing the activities of MCL-1i by designing molecules that target different moieties of MCL-1 or neutralize MCL-1 along with other prosurvival proteins of the BCL-2 family. Zhang et al. (39) demonstrated that it is possible to have a dual target for MCL-1; this is achieved by inhibiting MCL-1’s interaction with its proapoptotic BH3 counterparts and enhancing its ubiquitination and degradation. Zhang et al. discovered a hidden dynamic region—the Q221R222N223 (QRN) motif—in the BH3 domain of MCL-1 that controls MCL-1 ubiquitination and degradation. They reported that compound 5 binds to the H224 of MCL-1; thus, besides disrupting the MCL-1 interaction, it switches the BH3 domain towards a helical conformation, which facilitates MCL-1 ubiquitination and degradation. Similar observations have been made for maritoclax and UMI-77, which also are known to ubiquitinate and degrade MCL-1. In contrast, A-1210477 did not enhance *in vitro* ubiquitination of MCL-1 (39). Researchers have shown that lysine residue in the BH3 domain of MCL-1 can covalently bind an MCL-1i. NA1-115-7 has been shown to inhibit MCL-1 through a “covalent interaction between its 2 aldehyde functional groups and a lysine residue of the MCL-1 protein.” It triggered apoptosis in an MCL-1–dependent manner with no observed toxicity to peripheral blood cells or cardiomyocytes (145).

Dual MCL-1 and BCL-2 inhibitors have been designed to neutralize BCL-2 protein and partially inhibit MCL-1 to improve their safety and avoid MCL-1 upregulation (93). Both BCL-2 and MCL-1 share P2 as a common hot spot region and could be targeted by 2 novel compounds, IS20 and IS21, which induce apoptosis in melanoma and NSCLC cell lines. IS21 also decreased tumor growth in leukemic and melanoma mice models, comparable to ABT199 and ABT-263 (146). Additionally, Drennen et al. [148] developed an indazole-3-acylsulfonamide from a carboxylic acid core. The new compound inhibited both BCL-2 and MCL-1 and had a much lower affinity for BCL-xL. However, this compound was not tested in *ex vivo* or *in vivo* (147). Benzimidazole chalcone and flavonoid scaffold–derived bicyclic compounds were optimized to target both BCL-2 and MCL-1. These compounds exhibited significant cytotoxic activity against the AW13516 cell line in a caspase-dependent manner and displaced the BH3 binding partners of MCL-1 and BCL-2 (148). Dual inhibition of MCL-1 and BCL-xL has been proposed. However, dual-targeting inhibitors do not appear to be in clinical evaluation because of the increased hepatotoxicity seen when both MCL-1 and BCL-xL proteins are co-targeted systemically (149). Furthermore, the thrombocytopenia associated with BCL-xL inhibition may limit the use of MCL-1/BCL-xL dual inhibition (150, 151).

Although none of the direct inhibitors of MCL-1 have been successful in the clinic so far, targeting MCL-1 has remained attractive strategy. New MCL-1i patents for innovative agents derived from new chemicals or featuring new scaffolds are being filed and granted (152).

## MCL-1 inhibitors combinations

### Combinations with other BCL-2 family protein inhibitors

**Figure 4:**
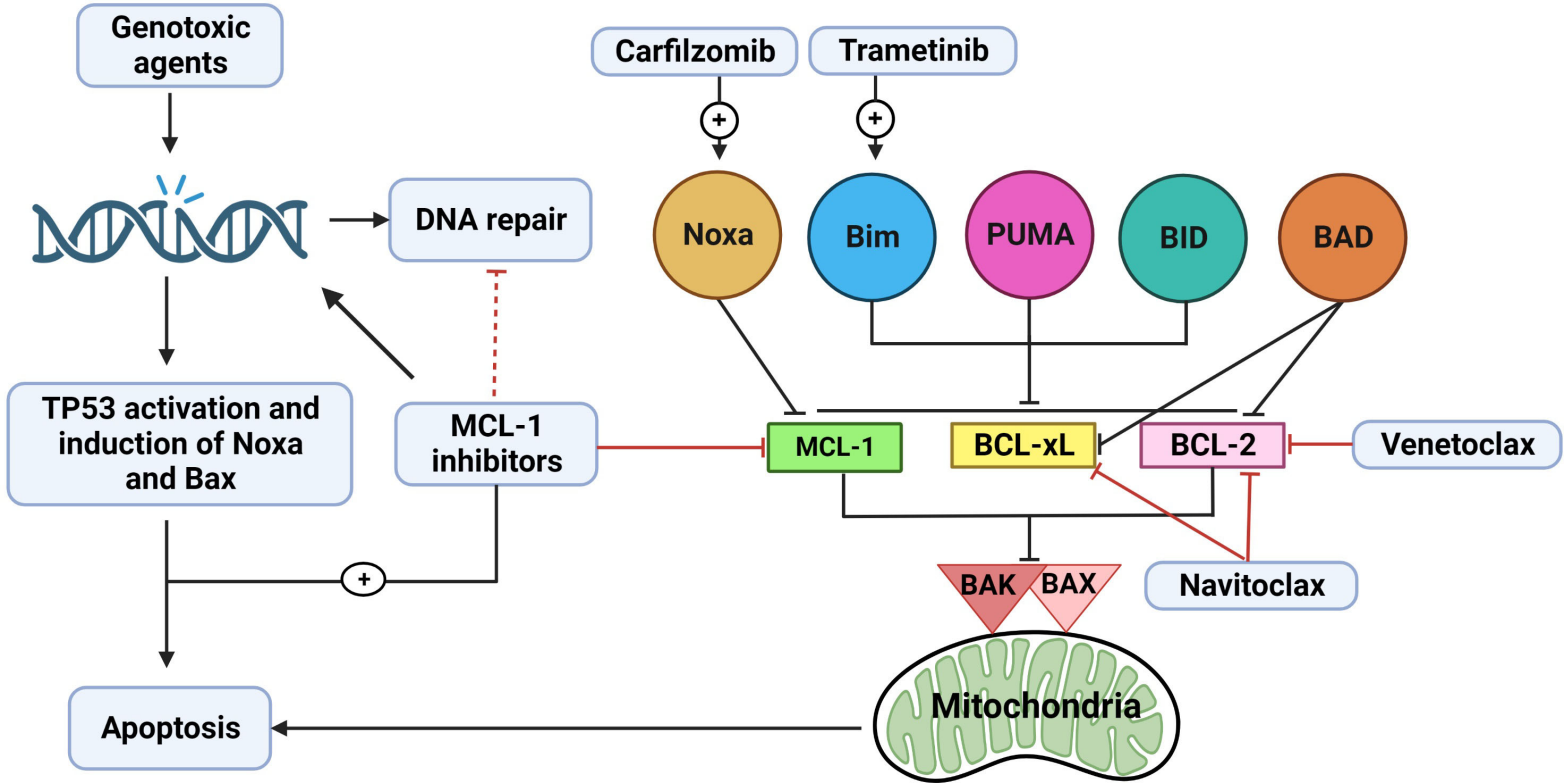
The rationale for combination therapy using MCL-1 inhibitors. Myeloid cell leukemia-1 (MCL-1), B-cell lymphoma (BCL)-2 extra large (BCL-xL), and BCL-2 prosurvival proteins cooperate to bind and inactivate BAX, BAK complex, and other proapoptotic BCL-2 homology (BH)3-only proteins (Noxa, BIM, PUMA, BID, and BAD). When one of these prosurvival proteins loses its function (e.g., from MCL-1 inhibition by MCL-1i), the other proteins (BCL-2 and BCL-xL) may at least partially compensate for this loss. Thus, combination therapy using MCL-1i with a BCL-2 inhibitor (e.g., venetoclax) or BCL-2/BCL-xL inhibitor (e.g., navitoclax) appears promising and has been shown to be effective. Another way to enhance the activity of MCL-1i is to increase the expression of BH3-only proteins. Trametinib can upregulate BIM, which can then bind to MCL-1, thus increasing dependency on MCL-1 protein and subsequently increasing sensitivity to MCL-1i. BIM can also bind to BCL-2 or BCL-xL, indirectly inactivating them. Carfilzomib can upregulate Noxa, which is a BH3-only sensitizer protein that binds to MCL-1, leading to more BH3-only activator proteins (BIM, PUMA, and BID) becoming available to bind to BAX/BAK and activate downstream apoptosis. Chemotherapy induces DNA damage with subsequent activation of TP53 and induction of BAX and Noxa. In addition, various chemotherapeutic agents can downregulate MCL-1. MCL-1i can synergize with chemotherapy by inducing more DNA damage, possibly affecting DNA repair, and neutralizing MCL-1. Effect of the clinically relevant MCL-1i (S63845, AMG-176 and AZD5991) on DNA repair mechanisms needs to be further explored.

Cells can hinder the response to MCL-1i by becoming dependent on multiple antiapoptotic proteins; in this way, the cells can compensate for one another (5, 114, 153). In such instances, administering combination therapy is the best option to achieve optimum antitumor activity (**Figure 4**). It has been shown that CLL is dependent on BCL-2 and has a robust response to venetoclax (154), while AML exhibits variable dependency on MCL-1 and BCL-2 (136). The variable dependency of AML on both BCL-2 and MCL-1 and the recent approval of venetoclax and azacytidine as a combination therapy for AML (these drugs were shown to be synergistic, as azacytidine was able to downregulate MCL-1) had led the use of combination therapies with BCL-2 inhibitors and MCL-1i for the treatment of AML. This approach is now being tested in clinical trials (5, 114). The combination of S63845 or AZD5991 with venetoclax has been found to be synergistic in AML and to overcome cytarabine resistance in the disease (155). The loss of TP53 impairs BAX/BAK activation, resulting in prolonged and sublethal targeting of either BCL-2 or MCL-1. Thus, intact TP53 function was found to be necessary to the action of BH3 mimetics, and the combination of MCL-1i and BCL-2 inhibitors may enhance long-term outcomes in aberrant-TP53 AML (156). CLL patient samples were found to be more dependent on BCL-2 than MCL-1, and low-dose venetoclax combined with AMG-176 has been shown to be additive or synergistic (130).

Another approach to enhancing the response to MCL-1i in cell lines that show less addiction to MCL-1 is to elicit an increased dependency on MCL-1 proteins in these cell lines by increasing the association between MCL-1 and proapoptotic proteins. This strategy was shown by Nangia et al. (157), who studied KRAS-mutant NSCLC cell lines that show some sensitivity to trametinib. The use of trametinib increased BIM expression (by stabilizing BIM) in NSCLC cells that are buffered by either BCL-xL or MCL-1; thus, the cells became dependent on both BCL-xL and MCL-1 protein. The use of navitoclax or AM-8621 synergized MEK inhibition in these cell lines. Prior BCL-xL inhibition increased MCL-1 dependence and enhanced sensitivity to MCL-1i, but not *vice versa*.

### Combinations with chemotherapeutic agents

In those cell lines that show sensitivity to MCL-1i, the combination of MCL-1i with standard-of-care chemotherapy or targeted therapy agents has been shown to be synergetic. MCL-1i were shown to be effective only in tumors dependent on MCL-1 protein. The growth of MM cells was found to rely on the presence of the MCL-1 protein, and they exhibited a more favorable response to MCL-1i and even superior to venetoclax (5, 114, 115). The MCL-1i were synergistic when combined with carfilzomib and dexamethasone, even in cell lines that were resistant to single-agent MCL-1i (114, 115). Interestingly, proteasome inhibitors upregulated Noxa protein, resulting in indirect inhibition of MCL-1 (158); this finding may explain the synergistic effect of combination therapy with MCL-1i and carfilzomib plus dexamethasone (159). A similar finding was reported in a murine double-hit lymphoma model, in which inhibiting MCL-1 protein synthesis by homoharringtonine and concomitant Noxa induction by bortezomib reduced tumor growth and increased survival significantly (160). Of note, the combination of AMG-176 with dexamethasone and carfilzomib in R/R MM is being studied in a clinical trial (NCT02675452).

Genotoxic agents induce DNA breaks with subsequent TP53, Noxa, and Bax upregulation and the induction of apoptosis (161). AZD5991 has been shown to induce DNA damage that is further increased when the drug is combined with cytarabine (162). MCL-1 depletion has been shown to induce genomic instability and impair DNA double-strand break repair (51). In addition, MCL-1 controls the switch between homologous recombination and nonhomologous end-joining DNA repair by binding to the Ku70/Ku80 dimers through its BH1 and BH3 domains; MCL-1 depletion reduces homologous recombination and favors nonhomologous end-joining repair. MI-223, which blocks the BH1 domain of MCL-1, impaired MCL-1–mediated homologous recombination DNA repair and sensitized cells to DNA-replication stress inducers (hydroxyurea and olaparib) (163). Studies are needed to explore the effects of the clinically relevant MCL-1i (S63845, AMG-176, and AZD5991) on DNA repair mechanisms. The potential effect of MCL-1 inhibition on DNA damage and DNA repair provides a rationale for combining MCL-1i with chemotherapy that upregulates TP53-mediated Noxa and Bax and depletes MCL-1, leading to apoptosis. It has also been shown that doxorubicin synergized with S63845 to induce apoptosis in BCP-ALL cell lines (164).

### Combinations with inhibitors of the MAP kinase pathway

The synergism of ERK1/2 inhibition with MCL-1i has also been reported in melanoma, in which AZD5991 synergized with ERK inhibition and delayed the development of BRAFi/MEKi resistance, thereby improving “the efficacy of an ERK1/2 inhibitor in a model of acquired BRAFi + MEKi resistance” (128). Similarly, the combination of MCL-1i and trametinib was also synergistic in AML cell lines and in 6 of 12 primary patient samples. Interestingly, it has been reported that the cell lines that had higher levels of MCL-1 protein were more susceptible to 50-nM S63845 than were cell lines with lower levels of MCL-1 protein. suggesting that MCL-1 and MEK1/2-protein expression levels may predict responses to S63845 and trametinib, respectively (165). Ulixertinib, another ERK1/2 inhibitor, is highly synergistic in rhabdomyosarcoma *in vitro* and *in vivo* through upregulation of the proapoptotic proteins BIM and BMF (166).

## Biomarkers of response and resistance mechanisms of MCL-1 inhibitors

High BAK expression and low BCL-xL expression predicted sensitivity to AM-8621 and ANJ810, while high BCL-xL expression predicted resistance to AM-8621 (114, 167). Response to S63845 inversely correlated with the expression level of *BCL2L1 gene* (5). MCL-1 protein level and mRNA expression correlated poorly with response to MCL-1i (5, 66, 114). Recently, some studies have revealed that the MCL-1/BCL-xL ratio predicted synergistic response to either AZD5991 or navitoclax in combination with an ERK1/2 inhibitor (128, 168). Current methods to predict the sensitivity to MCL-1i and dependency on MCL-1 protein include dynamic BH3 profiling or ex-vivo incubation by MCL-1i and observing the response (66, 127, 169). Clinical successes, genomics and multiomics data from MCL-1i clinical trials may provide clues to biomarkers of response.

It is well known that stromal microenvironment can provide resistance to the action of MCL-1i (79, 170, 171). Although the mechanism is not fully understood, the overexpression of MCL-1 and BCL-xL, in mantle cell lymphoma and CLL, is thought to mediate this resistance. Sensitivity can be restored upon the use of a BCL-xL inhibitor (173) or by AT-101, a pan-BCL-2 prosurvival protein inhibitor (172).

Cell lines resistant to MCL-1i has been used to identify mechanisms of resistance. Wang et al. (174) generated Mino and SUDHL cell lines resistant to MCL-1i (10× IC_50_). Compared to the parental cell lines, the resistant cell lines showed a higher level of BCL-2 protein and a slight increase in BCL-xL. Knocking down BCL-2 restored sensitivity to the MCL-1i S63845, confirming a role for BCL-2 in mediating resistance to MCL-1. Further kinome and transcriptome analysis revealed higher activity for the MEK, ERK, and BCR pathways, possibly impacting BCL-2. The resistant cell lines were more sensitive to MEK/ERK inhibition, further confirming a role for MEK and ERK pathways in the modulation of the BCL-2 family proteins.

In DLBCL cell lines, the loss of TP53 or BAX conferred resistance to AZD5991. The knockout of TP53 decreased BAX and PUMA expression, and BAX overexpression in TP53-deficient cell lines restored sensitivity to AZD5991, suggesting that a functional TP53 mediates sensitivity to AZD5991, at least partially, through BAX (175). Also, lower levels of BCL-2 and BCL-xL correlated with a higher sensitivity to S63845 in the HH cell line. Strikingly higher levels of BCL-W were found in the S63845-resistant MyLa and SeAx cell lines (176).

Bolomsky et al. (177) generated 2 myeloma cell lines (OPM2-R and KMS12BM-R) resistant to the MCL-1i S63845 to study MCL-1i resistance mechanisms. The resistant cell lines showed resistance to other MCL-1i (AZD5991 and AMG-176) and heterogenous modulation of cell type specific BCL-2 family proteins. BAK, BAX, and BIM were downregulated in the OPM2-R cells, MCL-1 and BCL-2 were upregulated in the KMS12BM-R cells, and there were no detectable alterations in the protein levels of the other BCL-2 family members (BAD, BID, Noxa, and PUMA). In addition,“high-throughput drug screening (n = 528 compounds) indicated alternative BH3 mimetics as best combination partners for MCL-1i in sensitive and resistant cells, particularly with BCL-xL inhibitors”.

Although BCL-2 mutations are known to drive resistance to venetoclax in CLL by inhibiting the binding of venetoclax to its target (178), very limited data are available regarding the potential mutations in MCL-1 that may hinder the activity of MCL-1i. Chen et al. (**179**) examined the frequency of mutations in BCL-2 family proteins in 982 MM patients (NCT0145429) and found that BCL-2 family protein mutations were generally rare. Interestingly, 10 patient samples were found to harbor MCL-1 mutations at baseline. These mutations were missense mutations in the N-terminal region (G32R; n = 1), in the PEST domain (n = 4; V140I, P142S, E149Q, and E173K), in an uncharacterized region between the PEST and BH1 domains (L186F), in BH1 (V249L and L267V), and within the BH3 (N223S and R214Q) domains. To functionally characterize the impact of these mutations, WT MCL-1 and mutant MCL-1 plasmids were overexpressed in an ALL murine cell line. The overexpressed human MCL-1 can replace the murine MCL-1 in this cell line. In cells with the V249L, N223S, and R214Q mutations, S63845 upregulated MCL-1, disrupted the MCL-1–BIM interaction, and showed a similar sensitivity to that in the WT MCL-1 cells. In contrast, the L267V mutation was resistant to both S63845 and AZD5991; in these cells, the MCL-1– BIM interaction was not disrupted, despite MCL-1 upregulation. This finding suggests that the L267V mutation did not hinder the binding of MCL-1i to MCL-1 protein, but that it blocked its ability to disrupt MCL-1’s interaction with BIM (179). Additional investigations are needed to assess clinical impact of these mutations. Also, MM cell lines with acquired carfilzomib resistance showed cross resistance to AZD5991, S63845, and A-1210477, but not to AMG-176. Immunoblot analysis revealed the upregulation of MDR1 protein, suggesting drug efflux as a mechanism of resistance to MCL-1i. The use of MDR1 inhibitors (tariquidar or verapamil) restored the sensitivity of these resistant cell lines to the action of MCL-1i (177). While some molecules are being identified for further studies of resistance, additional studies are needed.

In AML, c-Myc levels negatively correlated with the half-maximal effective concentration of AZD5991 in cell lines and primary patient samples. In the MOLM-13 and MV4-11 cell lines, which are resistant to AZD5991, the c-Myc and MCL-1 protein levels were upregulated. Targeting c-Myc by 10058-F partially overcome resistance to AZD5991 in resistant cell lines (180). A summary of predictive biomarkers and mechanisms of resistance is provided in **Table 2**.

**Table 2:** Summary of predictive biomarkers of response and mechanisms of resistance to MCL-1i.

	Biomarkers of response	Mechanisms of resistance
Identified	Biomarkers of sensitivity: -Increased BAK expression (114) -Increased cyclin D1 expression (181) -Increased MCL-1/BCL-xL expression (128, 168) -Low BCL-xL expression (167) Biomarkers of poor response: -High expression of BCL-xL (114), BCL-2 (114), or BCL-2L1 (5)	-Increased dependence on other BCL-2 family proteins like BCL-xL (173), BCL-2 (174, 177), or BCL-W (176) -Loss of TP53 or BAX (175) -L267V MCL-1 mutation (179) -Drug efflux through MDR1 (177) -Increased c-Myc expression (180)
Future directions	Determine the role of genetics (pharmacogenomics and MCL-1 mutations) and epigenetic mechanisms in modifying the response to or driving resistance to MCL-1i.	

BCL-2, B-cell lymphoma-2; BCL-xL, B-cell lymphoma extralarge; MCL-1, myeloid cell leukemia-1; MCL-1i, myeloid cell leukemia-1 inhibitor.

## MCL-1 inhibitors and challenges in the clinic

There are 2 major issues to be overcome regarding the clinical application of current MCL-1i. First, reports of MCL-1i cardiotoxicity are alarming and raise safety concerns. Second, researchers must determine how to fit MCL-1i into treatment algorithms to identify the patients likely to benefit from the drugs.

Cardiotoxicity appears to be a class effect of MCL-1i. The FDA put both AMG-397 and AZD5991 on hold because of concerns regarding cardiovascular adverse effects. The incidence of cardiotoxicity was low, the number of patients enrolled in the study was small, and the exact nature of the cardiotoxicity was not known. The work on AMG-397 is now stopped, while a related compound, AMG-176, is being tested in a phase 1 clinical trial. (https://ashpublications.org/ashclinicalnews/news/4765/FDA-Places-Trials-of-MCL-1-Inhibitor-on-Clinical?searchresult=1). Similarly, AZD5991 was put on clinical hold after reporting an asymptomatic elevation of laboratory cardiac parameters in one patient with multiple comorbidities that occurred in the AZD5991 and venetoclax combination arm.

The mechanism of MCL-1i–mediated incident cardiotoxicity is not very clear. MCL-1 has high expression in the myocardium and is essential for maintaining cardiac homeostasis and inducing autophagy in the heart. It has been shown that “cardiac-specific deletion of MCL-1 in mice” led to mitochondrial dysfunction, impaired autophagy, hypertrophy, and cardiomyopathy with distorted ultrastructure of disorganized sarcomeres and swollen mitochondria (182–184). Interestingly, concomitant BAX/BAK knockout in these mice models largely rescued the lethality and impaired cardiac function but not the ultrastructure changes of the mitochondria caused by the MCL-1 deletion (183). Also, double knockout of MCL-1 and cyclophilin D, which controls the mitochondrial permeability transition pore, extended survival and delayed the progression to heart failure (184). These data may indicate that the cell death associated with MCL-1 deletion contributes to the observed cardiac dysfunction, independently of mitochondrial dysfunction. Although the authors did not specifically study the effect of this mitochondrial dysfunction on the MCL-1 deletion induced cardiomyopathy, they predicted that the observed mitochondrial dysfunction may also contribute to the observed cardiotoxicity, owing to the importance of mitochondria to the cardiac function (185). Similarly, Perciavalle et al. (46), showed that MCL-1 deletion in murine embryonic fibroblast and hepatocyte distorted mitochondrial morphology with abnormal cristae and defective electron transport system. However, the direct effects of MCL-1i are different because MCL-1i upregulate, rather than deplete, MCL-1 (5, 114, 115). It is not known whether the cardioprotective function of MCL-1 depends on MCL-1’s BH3 domain or not. S63845 has been shown to disrupt cytoskeleton formation, mitochondrial morphology and dynamics in “cardiomyocytes derived from human-induced pluripotent stem cells”, leading to overall poor cardiomyocyte performance (186). These effects have been seen with high doses of the drug and/or prolonged periods of treatment. Besides, S63845 decreased cardiomyocyte viability in a dose dependent manner, supporting the dependency of the cardiomyocyte on MCL-1 for survival. Treating the hiPSC-CMs with 100 nM S63845 for two weeks impaired cardiomyocyte beating with mitochondrial dysfunction and impaired calcium influx, despite live cells, indicating that the impaired cardiomyocyte beating might be independent of cell death. Again, the mitochondrial dysfunction and decreased calcium influx was more prominent with treatment with S63845 and AZD5991 as compared with AMG-176. The use of the necrosis inhibitor IM-54, but not the caspase inhibitor QVD, rescued the cell death induced by S63845. Similarly, in Mec1 and HG3 CLL cell lines, AZD5991 was shown to decrease oxygen consumption rate (OCR), decrease ATP production, and increase mitochondrial oxygen reactive species, suggesting mitochondrial dysfunction (129). Organized mitochondrion is required for adequate cardiac function (185) through regulating metabolic bioenergetics, calcium flux and modulating cardiac contraction (187, 188). Its dysfunction has been linked to different cardiovascular diseases including cardiomyopathy and arrhythmias (189–191). To circumvent these side effects, researchers are working to identify new MCL-1i with low potential toxicity. ANJ810, for example, is a new potent MCL-1i with a short half-life and rapid systemic clearance; thus, it provides short-term inhibition of MCL-1 and has a low potential for cardiotoxicity (167).

Noxa downregulation provides an alternative mechanism of MCL-1i-mediated cardiotoxicity. The MCL-1i S63845 (141), AZD5991, and AMG-176 (140) downregulate Noxa protein upon disruption of their interaction with MCL-1 protein. This raises the question of whether the cardiac toxicity of MCL-1i is related to the targeting of MCL-1’s BH3 domain or to the downregulation of Noxa. Noxa downregulation has been shown to mediate phenylephrine-induced cardiac hypertrophy. Also, rapamycin has been shown to inhibit cardiomyopathy by promoting autophagy through beclin-1 and Noxa (192). Autophagy is important for the heart, and its dysregulation mediates several drug-induced cardiotoxicities, such as those resulting from daunorubicin treatment (193). Like MCL-1, Noxa can regulate autophagy (194, 195); therefore, it is important to explore the impact of MCL-1i-induced Noxa downregulation. An understanding of this downregulation may open the door for new combination strategies that maintain Noxa levels and could potentially enhance the efficacy and safety of MCL-1i.

In CLL cell lines, MCL-1i have been shown to induce a transient decrease in Mule expression that is associated with a compensatory increase of Mule at later time points (140). However, it is not known if similar changes occur in cardiomyocytes. Mule also has been shown to have a cardioprotective role against oxidative stress, and its deletion leads to cardiomyopathy (196).

**Figure 5:**
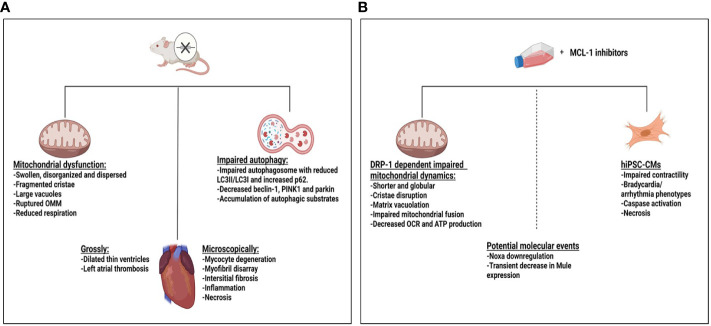
Effect of myeloid cell leukemia-1 (MCL-1) deletion or MCL-1 inhibitors on the heart. **(A)** Mcl-1 knockout in a mouse model led to dilated cardiomyopathy with thin ventricle walls and atrial thrombus, features of impaired cardiac hemodynamics. Ultrastructure changes in the mitochondria, myocyte degeneration, fibrosis, inflammation, and necrosis were observed. Mitochondrial dysfunction and impaired autophagy may have contributed to the observed toxicity. **(B)** Treating hiPSC-CMs with S68345 induced dynamin-related protein 1 (DRP-1–dependent mitochondrial dysfunction, cytoskeleton disruption, and impaired/irregular beating of the cardiomyocytes. The potential role of Noxa and Mule downregulation on cardiotoxicity needs to be further explored. ATP, adenosine triphosphate; OCR, oxygen consumption rate; OMM, outer mitochondrial membrane.

MCL-1i induced cardiotoxicity may be related to the high affinity of MCL-1i for MCL-1 protein, at least in part. A study of mouse lymphoma models knocked in with human MCL-1 showed that S63845 was efficacious at tolerable doses. “However, the maximum tolerated dose was lower in huMcl-1 mice than in the control group (138, 197), suggesting that S63845 toxicity depends on its immediate target, MCL-1, although the precise mechanism is unknown”. As discussed earlier, MCL-1 protein is engaged in various non-antiapoptotic functions, particularly autophagy, oxidative phosphorylation, and mitochondrial bioenergetics. It is not known whether these functions depend on the BH3 domain of MCL-1 protein or not. Thus, further studies to assess how MCL-1i can affect the non-antiapoptotic function of MCL-1 are needed to better understand the cardiotoxicity associated with MCL-1i. In summary, data regarding MCL-1i–induced cardiotoxicity are limited. Multiple factors may contribute to the observed cardiotoxicity, including the very high affinity of MCL-1i for MCL-1, high doses of and prolonged exposure to MCL-1i, mitochondrial dysfunction, impaired autophagy, and the induction of cell death. Accumulating evidence suggests that cardiotoxicity be related to the cardiomyocyte cell death and or/mitochondrial dysfunction and impaired calcium influx. Mitochondrial dysfunction is predicted to contribute to the observed cardiotoxicity either through the impaired bioenergetic metabolism, ATP production, impaired calcium influx, increased ROS production and/or through potentiating cell death. Current efforts to circumvent these unwanted events include designing inhibitors that provide partial inhibition of MCL-1 (dual MCL-1 and BCL-2 inhibitors) and MCL-1i that provide short-term inhibition of MCL-1 (e.g., ANJ810). Future studies are needed to explore the role of Noxa and Mule downregulation associated with the use of MCL-1i and observed cardiotoxicity and to determine the effect of MCL-1i on other non-antiapoptotic functions of MCL-1. The effect of MCL-1 deletion or MCL-1 inhibition on the heart is summarized in **Figure 5**.

It is important to determine how to fit MCL-1i into treatment algorithms for optimal benefits to patients. It appears that, compared to solid tumors, hematologic malignancies show preferential sensitivity to MCL-1i (114, 115). Preclinical data indicate that MCL-1i are efficacious in cell lines and tumors that are dependent on MCL-1 for survival. The classic example of this is MM, in which MCL-1 appears to act as a gatekeeper against apoptosis. Accordingly, there is a good chance for MCL-1i to be successful in patients with MM (114). AML, on the other hand, shows dual or heterogenous dependency on BCL-2/MCL-1. In addition, MCL-1 appears to be a major driver of resistance to venetoclax (136, 198), underscoring the value of the combination of MCL-1i and venetoclax. In CLL, it is clear that the disease is mainly dependent on BCL-2 and that patients benefit the most from the use of venetoclax alone (199). However, an approach to testing combination therapies with venetoclax and MCL-1i is needed. In fact, declines in MCL-1 have been achieved *via* ibrutinib treatment followed by venetoclax treatment; this combination therapy appears to be successful in CLL (200, 201) and MCL. Future studies are needed to determine the optimum combination therapy approaches involving MCL-1i. It is feasible that the transient use of MCL-1i in mechanism-based combinations may benefit patients without untoward cardiotoxicity.

## Conclusions and future considerations

Laboratory cell line data, murine model systems, and clinical observations clearly underscore MCL-1 as a therapeutic target for many cancers. Although 6 MCL-1i that directly neutralize MCL-1’s function are being tested in phase 1 clinical trials, our knowledge about the use of MCL-1i in the clinic is currently limited and depends upon how successful the clinical trials are. Optimizing and designing new potent and specific MCL-1i is urgently needed in light of the emerging role of MCL-1 in tumorigenesis and therapeutic resistance. Identifying biomarkers of response and resistance will guide us to better uses of MCL-1i in the clinic. In addition, gaining a deeper understanding of the effects of MCL-1i on the non-antiapoptotic function of MCL-1 may help improve the safety profiles of MCL-1i. Efforts from chemists and pharmaceutical interests, the enthusiasm of scientists to carve out optimal MCL-1i usage and combination strategies, and clinical endeavors and observations are at their peak levels and will help make MCL-1 a clinical target.

## Author contributions

ST researched the literature and outlined and wrote the review. NT reviewed the manuscript, created the figures, and wrote the figure legends. AS and VG supervised the project, researched the literature, reviewed, wrote, and edited the manuscript, and outlined the review. All authors contributed to the article and approved the submitted version.
